# Is inpatient monitoring necessary in pediatric patients with comorbidities following routine dental procedures? A retrospective analysis

**DOI:** 10.1007/s10006-026-01603-y

**Published:** 2026-07-18

**Authors:** Max Lukas Linderkamp, Efthymios Papazacharias, Madlen Sophie Eich, Jan Gaessler, Pascal Eber, Alexander Rahman, Philipp Jehn, Fritjof Lentge, Michael-Tobias Neuhaus, Nils-Claudius Gellrich, Philippe Korn

**Affiliations:** 1 Department of Oral and Maxillofacial Surgery, Hannover Medical School, Carl- Neuberg-Str. 1, 30625 Hannover, Germany; 2 Department of Conservative Dentistry, Periodontology and Preventive Dentistry, Hannover Medical School, Carl-Neuberg-Str.1, 30625 Hannover, Germany

**Keywords:** Dental surgical procedures, Anesthesia, general, Pediatrics, Perioperative complications, Child, medically complex

## Abstract

**Purpose:**

Children and young adults with severe pre-existing conditions frequently require dental treatment under general anesthesia (GA) and are commonly scheduled for postoperative inpatient surveillance due to presumed elevated anesthetic or surgical risks. Although this approach prioritizes safety, it may contribute to prolonged or unnecessary hospitalization, increased healthcare costs, and potential psychological burden, particularly in patients with neurodevelopmental or congenital disorders. In light of the reportedly low incidence of severe complications following dental treatment under GA, this study aims to evaluate the necessity of routine inpatient admission in pediatric patients (< 18 years of age) with comorbidities and to assess the frequency and severity of perioperative complications.

**Methods:**

All pediatric patients with pre-existing conditions who underwent planned inpatient surgical dental treatment under GA at the Department of Oral and Maxillofacial Surgery at Hannover Medical School between 2014 and 2025 were included. The indications for inpatient treatment, anesthesiological parameters such as the American Society of Anesthesiologists (ASA) classification, and severe perioperative complications (corresponding to grade ≥ 2 of the Clavien-Dindo classification) were analyzed. It was assessed whether the ASA classification or the type of pre-existing condition significantly influenced the occurrence of perioperative complications.

**Results:**

A total of 199 patients were analyzed. Patients who underwent surgery were mainly categorized as ASA II (*n* = 80, 40.2%) and III (*n* = 102, 51.3%), and mostly presented with cardiovascular (*n* = 68, 34.2%) or hematological/oncological preconditions (*n* = 47, 23.6%). Perioperative complications occurred in 8 cases (4.2%); of those, 6 were intraoperative (3.1%), and 2 were postoperative (1.0%). The probability of perioperative complications did not correlate with ASA classification or pre-existing conditions (*p* [Fisher] = 0.166, and *p* [Fisher] = 0.571, respectively). Age significantly influenced the probability of complications (U = 451.00, *p* = 0.050), indicating that older patients were more likely to experience complications than younger patients.

**Conclusion:**

As postoperative complications occur very rarely, even in patients with pre-existing conditions, inpatient surveillance should warrant thoughtful consideration to liberate hospital capacities. Interdisciplinary pre-, intra-, and postoperative assessment of the need for inpatient surveillance should ensure patient safety and provide further insights into risk factors for peri- and especially postoperative complications. The authors recommend validating risk scores and algorithms for decision-making regarding inpatient surveillance.

## Objective

Children and young adults suffering from severe pre-existing conditions represent a particularly vulnerable group in dental medicine. While in most cases, standard dental procedures can be performed under local anesthesia, young patients with neurodevelopmental, congenital, or extensive past medical history are unable to tolerate treatment under standard local anesthesia settings and therefore require therapy under general anesthesia (GA). GA for dental therapy is required significantly more often in young patients presenting with pre-existing conditions (87.7% vs. 63.3% in healthy controls; [1]). The most common reason for dental treatment is caries (up to 91%; [2]), which, in turn, most often results in tooth extraction and stainless-steel crowns, but may also involve restorative dentistry, such as pulp treatment and direct restorations [3]. While treatment under GA ensures safety, adequate pain control, and procedural feasibility, resulting in higher-quality treatment [3] and thereby offering a positive effect on oral health-related quality of life [4], pre-existing conditions often appear to confer high-risk profiles for postoperative surveillance, leading to inpatient surveillance in many cases, sometimes with long inpatient stays.

Inpatient stay is indicated by anesthesiologists and surgeons for internal medicine reasons, such as cardiovascular or pulmonary preconditions, neurodevelopmental disorders, and due to the risk of postoperative hemorrhagic complications, especially in cases with innate coagulation problems. Recent studies have shown that pediatric patients with two or more complex chronic conditions have a higher likelihood of hospital admission, and further readmission for dental treatment and experience significantly longer inpatient stays [5].

While this strategy offers high patient and physician safety, it might lead to overtreatment, such as excessive or prolonged inpatient stay, which in turn may lead to hospitalization and show negative psychological effects, especially in patients with congenital development disorders or in patients who, independent of dental treatment, do have extensive histories of hospitalization [6]. Opposed to the wish for safety are extremely rare cases of potentially life-threatening complications following dental restoration, either using GA or sedation, at a rate of 0.5-1,4 per 1,000,000 patients [7, 8].

Not least due to demographic developments and limited healthcare resources, healthcare expenses should be based on evidence and kept as low as possible without endangering patients’ safety. For this reason, this study aims to evaluate the actual necessity of postoperative inpatient admission, as perioperative complications may be exceptionally rare even in high-risk pediatric patients.

## Materials and methods

All patients under 18 years of age with pre-existing conditions who underwent planned surgical dental treatment in the Department for Oral and Maxillofacial Surgery at Hannover Medical School between 2014 and 2025 under GA were included and evaluated retrospectively. Patients who presented as emergencies for immediate surgery were excluded from the study. The decision for postoperative inpatient observation was made by the treating surgeons and anesthesiologists, based on the standard operating procedures (SOP) of their respective departments.

Data was obtained using the clinical information system. Sex, age, reason for surgery, general health, and pre-existing conditions were displayed, and the mean inpatient stay and surgery durations were evaluated. The American Society of Anesthesiologists (ASA) classification was used to stratify patients’ anesthesiologic risk, as it is part of the standard preoperative assessment strategy. The ASA classification underwent a major revision in 2014; the latest version was used to assess all patients.

The number and type of complications were reported, and whether their occurrence correlated with either the ASA classification or pre-existing conditions.

Severe events requiring further medical intervention (corresponding to grade ≥ 2 of the Clavien-Dindo classification) were included in the analysis (*major complications*). Standard postoperative side effects, such as swelling or pain, and minor complications, such as postoperative nausea and vomiting (PONV, corresponding to grade 1 of the Clavien-Dindo classification), were not included in the analysis. While the Clavien-Dindo classification is a postoperative system, intraoperative complications were classified accordingly.

Costs for procedures and inpatient stay were calculated using the diagnosis-related group (DRG) calculator based on the International Classification of Diseases, version 10, in the German modification (ICD-10-GM) and the Operation and Procedure Classification (Operationen- und Prozedurenschlüssel, OPS). Costs for outpatient procedures were calculated using the Uniform Value Scale (Einheitlicher Bewerungsmaßstab, EBM) and the Scale of Fees for Dental Services (Bewertungsmaßstab für zahnärztliche Leistungen, BEMA). Both systems employ a bundled payment structure, which incorporates costs for general anesthesia, staffing, and all necessary materials.

Statistical analyses were conducted using Microsoft Excel 2016 (Microsoft, Redmond, WA, USA) and IBM SPSS Statistics^®^ Version 30.0.0.0 (IBM, Armonk, NY, USA). Descriptive statistics were used to report basic epidemiologic information on the patient collective, percentages were rounded to one decimal place. Fisher’s exact test and the Mann-Whitney U test were used to determine whether a significant correlation existed. A p-value of ≤ 0.05 was considered statistically significant.

## Results

In total, 199 patients were treated at the Department of Oral and Maxillofacial Surgery at Hannover Medical School between 2014 and 2025 in an inpatient setting. Of those patients, 87 were female (43.7%), and 112 were male (56.3%), resulting in a male-to-female ratio of 1.29:1. The mean age was 6.91 years (*n* = 199, SD = 2.66, range: 1.70–12.90 years). The most frequently performed (surgical) procedure were tooth extractions (*n* = 188, 94.5%), either solely (*n* = 96, 48.2%), combined with a non-surgical dental restoration (*n* = 81, 40.7%), or combined with other dental-associated surgeries (*n* = 11, 5.5%), of which 6 were tooth extraction combined with abscess incision (3.0%). Minor procedures were non-surgical dental restoration only (*n* = 6, 3.0%), surgical exposure of an unerupted tooth (*n* = 2, 1.0%), incision of a dental abscess (*n* = 2, 1.0%), or the combination of non-surgical dental restoration and the surgical exposure of an unerupted tooth (*n* = 1, 0.5%). Most patients received surgical dental treatment for the first time (*n* = 143, 71.9%), whereas fewer had been treated more than once (*n* = 50, 25.1%).

### General health and pre-existing conditions

**Fig. 1:**
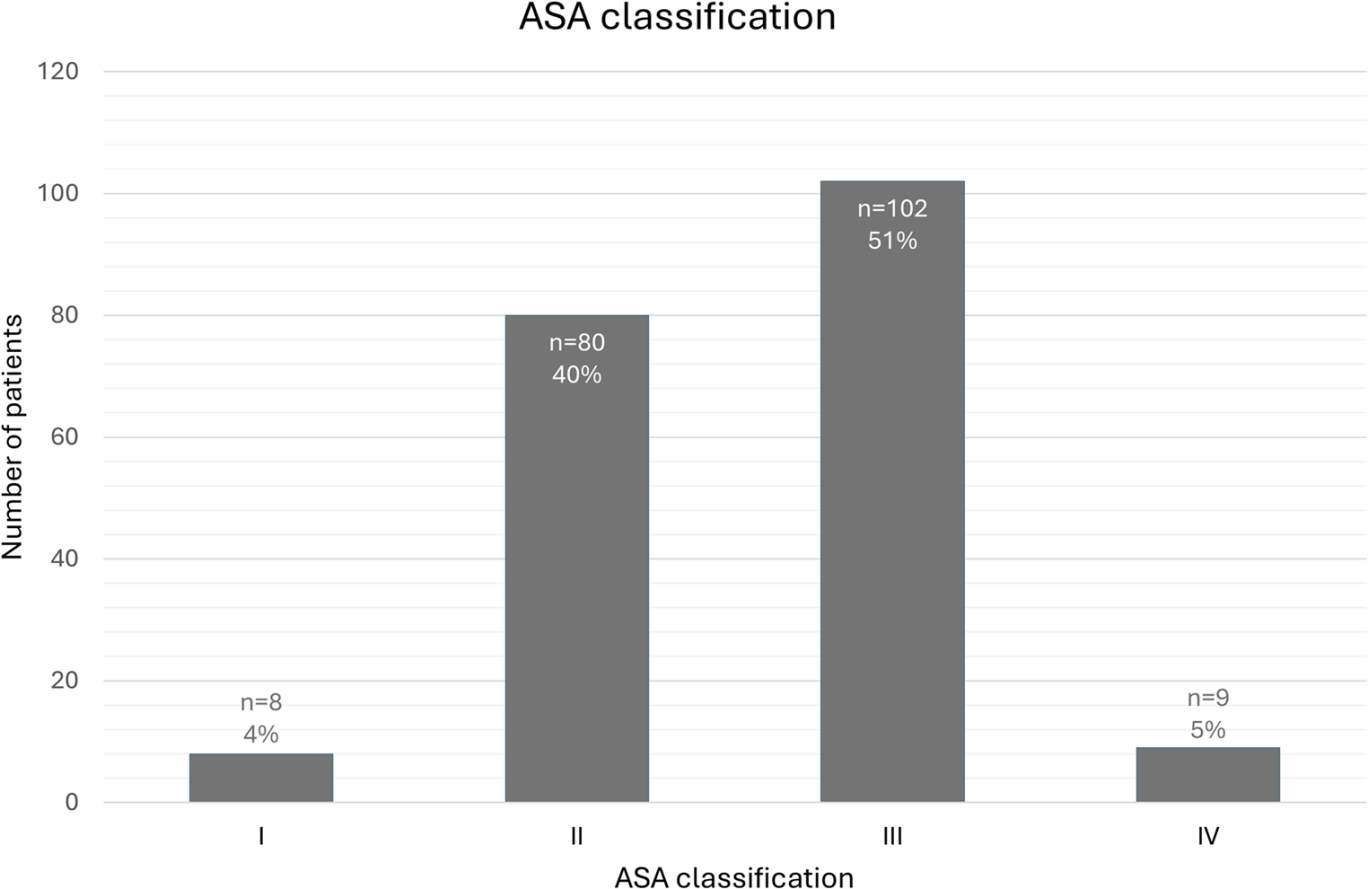
ASA classification of children with pre-existing conditions who underwent dental surgical restoration under general anesthesia between 2014 and 2025. The numbers in the columns indicate the number of patients (n) and the percentage (rounded)

**Fig. 2:**
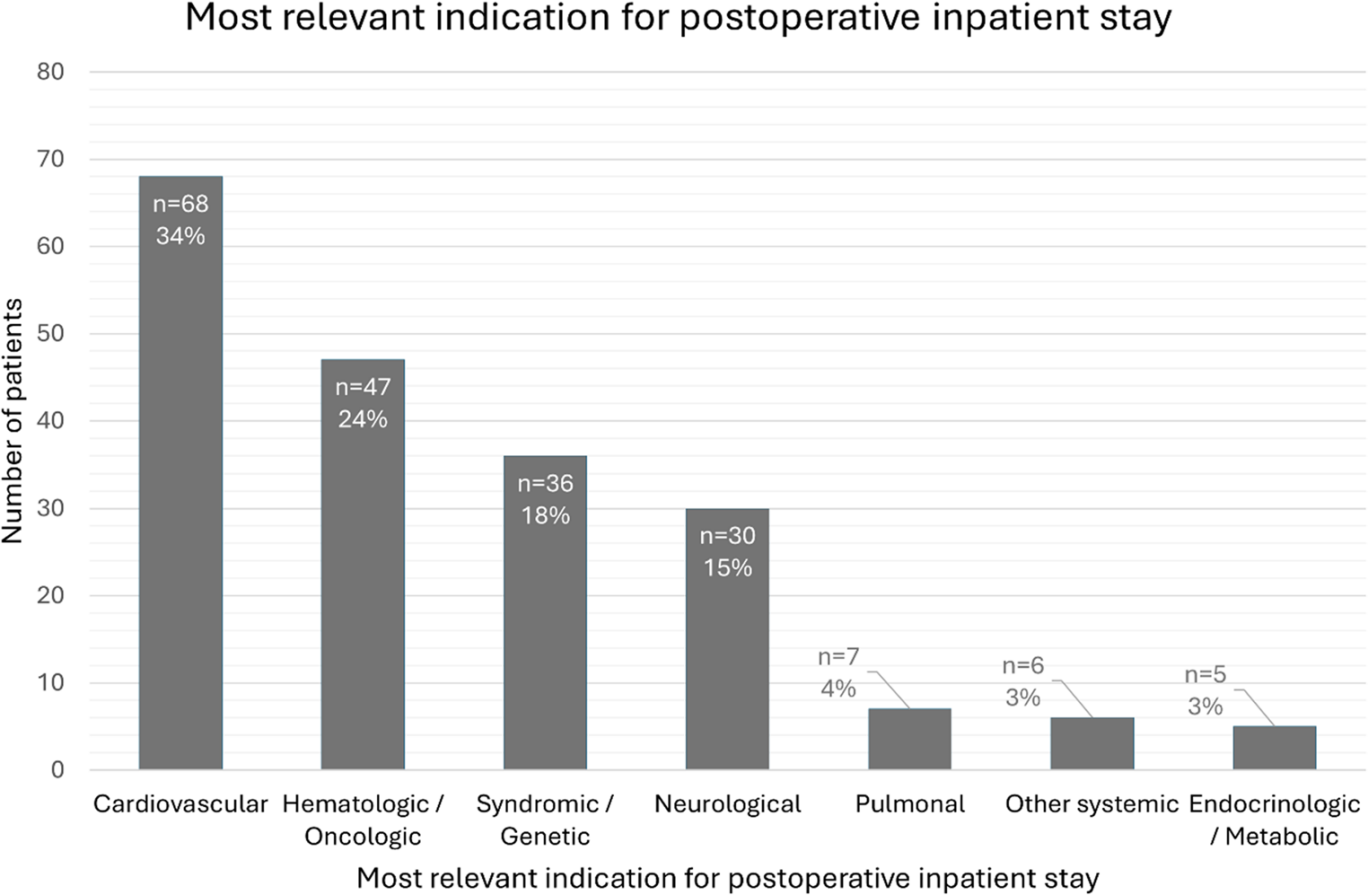
Most relevant pre-existing conditions that led to the indication for inpatient surveillance. The numbers in the columns indicate the number of patients (n) and the percentage (rounded)

Patients who underwent surgery were primarily classified as ASA II (*n* = 80, 40.2%) and III (*n* = 102, 51.3%), with fewer patients categorized as ASA I (*n* = 8, 4.0%) and ASA IV (*n* = 9, 4.5%; Fig. 1). The most relevant pre-existing conditions for postoperative inpatient surveillance were cardiovascular (*n* = 68, 34.2%) and hematological and oncological conditions (*n* = 47, 23.6%). Of those, 36 patients (18.1%) had hematologic pre-existing conditions, and 11 (5.5%) had oncologic pre-existing conditions. The distribution of pre-existing conditions most relevant for indicating inpatient surveillance is shown in Fig. 2.

### Duration

The mean duration of inpatient stay was 2.57 days (*n* = 199, SD = 1.51, range: 1–16 days). The extended 16-day hospitalization resulted from a catheter-related bloodstream infection, which required treatment during the same inpatient stay. Complete documentation of surgical and anesthesia times was available for 191 patients. Mean surgery time was 63.03 min (SD = 49.77, range: 4–269 min), and the mean anesthesia time was 109.10 min (SD = 62.03, range: 9–369 min).

### Complications

Perioperative complications occurred in 8 cases (4.2%); of those, 6 were intraoperative (3.1%), and 2 were postoperative (1.0%).

Intraoperative complications included self-limiting bradycardia (heart rate down to 45 beats per minute), laryngospasm with cessation of spontaneous ventilation, vomiting while emerging from anesthesia, a decrease in oxygen levels during anesthesia, the necessity for resuscitation because of bradycardia and laryngospasm, and the occurrence of ST-elevations and bigeminy.

Postoperative complications comprised oral hemorrhage, which had to be controlled by surgery under GA and atrial tachycardia with tachyarrhythmia absoluta.

In the group with hematologic pre-existing conditions, including patients with blood coagulation disorders, no post-operative complication occurred, especially no case of oral hemorrhage that required further (surgical) intervention.

The ASA classification showed no significant correlation with the probability of intraoperative (*p* [Fisher] = 0.111), postoperative (*p* [Fisher] = 0.586), or perioperative complications (*p* [Fisher] = 0.166, Table 1). Neither did the pre-existing conditions (*p* [Fisher] = 0.571).

**Table 1:** Correlation between ASA classification and the occurrence of perioperative complications

	Perioperative Complications			
	Yes [*n*, (%)]	No [*n*, (%)]	Total [*n*, (%)]	*p* [Fisher]
ASA classification				0.166
I	1 (12.5)	7 (3.7)	8 (4.0)	
II	2 (25.0)	78 (40.8)	80 (40.2)	
III	4 (50.0)	98 (51.3)	102 (51.3)	
IV	1 (12.5)	8 (4.2)	9 (4.5)	
**Total**	**8 (4.0)**	**191 (96.0)**	**199 (100.0)**	

There was no correlation between the duration of surgery or anesthesia and the occurrence of perioperative complications (U = 786.00, *p* = 0.724; U = 630.00, *p* = 0.505, respectively).

**Fig. 3:**
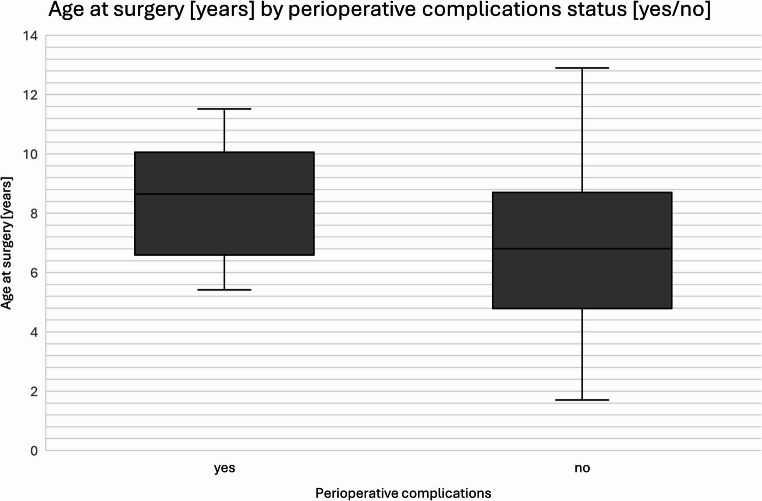
A box plot showing the age distribution among patients with perioperative complications (left) and without (right)

Age significantly influenced the probability of perioperative complications (U = 451.00, *p* = 0.050), indicating that older patients were more likely to experience complications than younger patients (patients with perioperative complications: mean age = 8.6 years, SD = 2.04; patients without perioperative complications: mean age = 6.8 years, SD = 2.66; Fig. 3).

The occurrence of complications, whether intraoperative, perioperative, or postoperative is not associated with prior inpatient stays (*p* [Fisher] = 0.650, *p* [Fisher] = 0.208, *p* [Fisher] = 0.066, respectively).

### Procedural costs

For the approximate, illustrative cost calculation, a sample case was designed based on the mean inpatient duration and the most frequently performed procedure. The most common case is a tooth extraction, with an average inpatient stay of 2.57 days. Since inpatient stays cannot be calculated in half-day increments, the estimate was based on a case involving a stay of 2–3 days. This equates to approximately 3,000–3,500€, depending on the federal state. In contrast, the same procedure in an outpatient setting would cost approximately 300–500€, depending on the extent of the procedure.

## Discussion

GA is a valuable tool for children and young adults with pre-existing conditions, as it allows safe and adequate dental treatment while also reducing the likelihood of psychological trauma associated with dental procedures [3]. Perioperative complications were rare (4.2%), and postoperative complications occurred in only 2 cases (1.0%). To the best of the authors’ knowledge, this study represents one of the largest cohorts to assess major complications following GA for dental treatment in children and young adults with pre-existing conditions. Most literature to date reports perioperative complication rates in healthy pediatric patients; however, rates of 1.1–3.2% in healthy or mixed cohorts are comparable to those observed in the present patient collective [9–11]. Given the low incidence of complications, GA and dental treatment in this so-called high-risk population can be considered safe [10].

Nevertheless, inpatient surveillance following GA is often indicated by anesthesiologists and surgeons for pediatric patients with pre-existing conditions. Considering the low postoperative complication rates observed in this study, such surveillance might constitute overtreatment.

However, predicting perioperative complications preoperatively remains challenging for clinicians. Accordingly, the present study observed no correlation between pre-existing conditions, the ASA score and the occurrence of complications.

Historically, decisions regarding postoperative inpatient monitoring were often influenced by the ASA classification. However, recent publications have criticized the ASA system - especially in pediatric patients - due to its subjective definitions and simplicity [12]. Originally designed for adults, the ASA classification may underestimate morbidity in pediatric patients [13] and demonstrates limited interrater reliability [14]. This may explain the lack of correlation between ASA classification and postoperative complications, highlighting its limitations for preoperative risk assessment in pediatric patients.

To overcome the limitations of the ASA classification and improve the prediction of perioperative morbidity and mortality in pediatric populations, several scoring systems have been developed [15]. Evidence suggests that incorporating intrinsic surgical risk, which is low in cases of dental surgery, improves the prediction of postoperative morbidity [16].

In this context, Nasr et al. introduced the Risk Assessment of Morbidity in Pediatric Surgery (RAMPS) score in 2020 to predict perioperative morbidity in children undergoing noncardiac surgery. This score integrates patient age, critical illness, chronic condition indicators (CCI), and intrinsic surgical risk (ISR) [17]. The RAMPS score demonstrated strong predictive ability (area under the curve [AUC] = 0.805; 95% confidence interval [CI], 0.795–0.816) and excellent internal and external validity (bias-corrected Nagelkerke R² = 0.21 and AUC = 0.783; 95% CI, 0.770–0.797, respectively). Ranging from 0 to 10, the RAMPS score stratifies morbidity risk as low (0–3; 3.4%, 95% CI 3.0-3.9), moderate (4–7; 11.6%, 95% CI 10.9–12.4), or high (8–10; 33%, 95% CI 30.7–35.3).

**Fig. 4:**
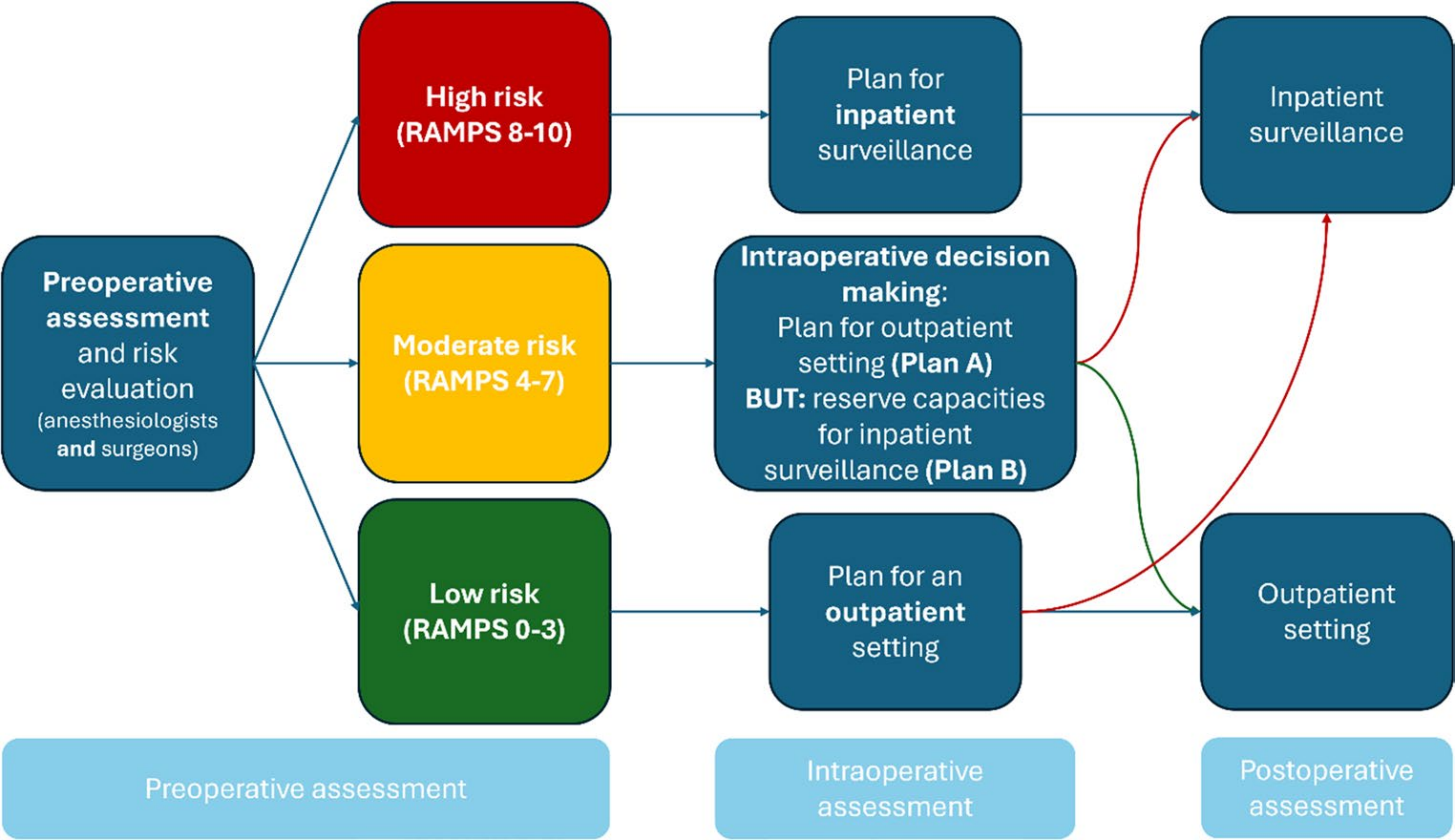
Proposed algorithm for validation to guide decision-making regarding postoperative inpatient surveillance in pediatric patients with pre-existing conditions. Risk assessment could be performed using the RAMPS score. We hypothesize that for low-risk patients (RAMPS score 0–3), an outpatient setting is appropriate, with escalation reserved for intraoperative or postoperative emergencies. In moderate-risk patients (RAMPS score 4–7), intraoperative decision-making regarding postoperative surveillance is recommended. This requires prior organization of inpatient capacity, although its use may ultimately not be necessary. For high-risk patients (RAMPS score 8–10), inpatient postoperative surveillance is recommended, even if GA and the surgical procedure are uneventful. However, this algorithm warrants further validation for this specific patient cohort.

To guide decisions regarding inpatient surveillance, we propose validating a preoperative algorithm based on the RAMPS score to identify patients at highest risk, while reserving the majority of decisions regarding postoperative inpatient surveillance for the intraoperative period (Fig. 4).

We recommend validating the RAMPS score, along with the algorithm presented in Fig. 4, to guide decisions on postoperative surveillance in pediatric dental surgery. Since dental procedures fall into the low-risk ISR category (first quartile), applying this stratification could allow more patients to be treated safely in an outpatient setting, consistent with Puri et al. [18]. This approach could ensure patient safety, enable selective postoperative monitoring, optimize hospital capacity, and significantly reduce healthcare costs, as outpatient procedures cost only a fraction of comparable inpatient treatments.

The present study also aimed to identify risk factors indicating the need for inpatient surveillance in pediatric patients. Interestingly, older patients were more susceptible to perioperative complications, contrasting with existing risk scores that typically identify younger patients as higher-risk [19, 20]. This finding may be due to the exclusion of patients under one year of age, as dental issues are uncommon before primary tooth eruption, and because of the rarity of complications (*n* = 8). Consequently, definitive clinical recommendations regarding the correlation between the occurrence of complications and the patients’ age should not be formulated until a larger patient cohort has been investigated.

As a retrospective study, some limitations exist. The study lacked a healthy control group; however, previous reports on healthy cohorts have documented no serious complications in 7,041 cases [9]. In certain instances, the rationale for inpatient stay was difficult to determine retrospectively, particularly when multiple pre-existing conditions were present. A prospective study would allow more precise risk stratification. Additionally, this study focused on major complications. Minor complications or events not requiring inpatient care, such as postoperative nausea and vomiting, were not captured. Literature research also revealed inconsistent definitions of “complication,” with some studies counting postoperative pain as a complication despite it being a normal physiological response to surgical tissue injury [2].

Additionally, the statistical power of this study is limited by the very small number of observed events. We therefore encourage other centers providing routine dental surgery care for children with pre-existing conditions to collect data, so that final recommendations can be derived from multicenter data. This would also allow for subgroup analyses to preoperatively screen and protect highly vulnerable patients, while allowing those with very low perioperative risk to be treated on an outpatient basis.

As the present study was conducted at a single German university hospital, the data inherently reflect only the specific structures and guidelines of the department’s SOPs and the German healthcare system. Consequently, indications for inpatient admission and follow-up care structures may differ across varying departments and international healthcare systems, leading to disparities in how postoperative admissions are handled and how complications are recorded and assessed. Regarding the German healthcare system, outpatient care was associated with substantially lower costs compared to equivalent inpatient admissions. However, emergency transfers to the hospital for unexpected complications during outpatient surveillance can partially offset these savings. Nevertheless, the results of the present study demonstrate that postoperative events were rare.

In summary, GA and the postoperative course in pediatric patients with pre-existing conditions are associated with very low complication rates. Therefore, routine postoperative inpatient surveillance should be reassessed, not least, in light of the psychological strain and economic costs. Decisions regarding inpatient care should be individualized, based on careful consultation with experienced anesthesiologists and surgeons [21], and supported by effective interprofessional communication [22]. Optimal dental treatment under GA should be planned in an interprofessional setting, with anesthesiologists contributing to the determination of treatment extent while balancing procedural benefits against anesthesia-related risks [23]. It is important that in the whole decision-making process any clinician involved retains the authority to recommend inpatient care if uncertainty exists. Further research is warranted, as previously recommended by Messieha et al. [24], to identify high-risk patients who may benefit most from postoperative inpatient monitoring. Validating the RAMPS score and the proposed algorithm could lead to a reassessment of routine postoperative surveillance, liberating hospital capacity while ensuring safe dental treatment under GA for children with pre-existing conditions. The use of this algorithm could address anesthesiologists’, surgeons’, and parents’ desire for a highly safe treatment, meet children’s need for a sense of security and a familiar environment, and help limit the currently rising costs of the healthcare system.

## Data Availability

The data that support the findings of this study are not openly available due to reasons of sensitivity and are available from the corresponding author upon reasonable request. Data are stored in controlled-access data storage at MHH.
